# Emerging Infectious Determinants of Chronic Diseases

**DOI:** 10.3201/eid1207.060037

**Published:** 2006-07

**Authors:** Siobhán M. O'Connor, Christopher E. Taylor, James M. Hughes

**Affiliations:** * Centers for Disease Control and Prevention, Atlanta, Georgia, USA;; † National Institutes of Health, Bethesda, Maryland, USA;; ‡ Emory University, Atlanta, Georgia, USA

**Keywords:** Infectious disease, chronic disease, infectious etiology, infection, microbial threats, bacterium, fungus, parasite, virus, causality, perspective

## Abstract

Evidence now confirms that noncommunicable chronic diseases can stem from infectious agents. Furthermore, at least 13 of 39 recently described infectious agents induce chronic syndromes. Identifying the relationships can affect health across populations, creating opportunities to reduce the impact of chronic disease by preventing or treating infection. As the concept is progressively accepted, advances in laboratory technology and epidemiology facilitate the detection of noncultivable, novel, and even recognized microbial origins. A spectrum of diverse pathogens and chronic syndromes emerges, with a range of pathways from exposure to chronic illness or disability. Complex systems of changing human behavioral traits superimposed on human, microbial, and environmental factors often determine risk for exposure and chronic outcome. Yet the strength of causal evidence varies widely, and detecting a microbe does not prove causality. Nevertheless, infectious agents likely determine more cancers, immune-mediated syndromes, neurodevelopmental disorders, and other chronic conditions than currently appreciated.

## Abstract 2

Infectious agents, often through complex systems, likely determine more chronic diseases than is currently appreciated.

Infectious agents have emerged as notable determinants, not just complications, of chronic diseases. Not infrequently, infection may simply represent the first misstep along a continuum from health to long-term illness and disability. Preventing or treating infection or the immune response to infection offers a chance to disrupt the continuum, avoiding or minimizing a chronic outcome. To capitalize on these opportunities, clinicians, public health practitioners, and policymakers must recognize that many chronic diseases may indeed have infectious origins.

A diverse spectrum of agents, pathways, outcomes, and co-factors characterize the already well-established causal associations. Together, this group affects all populations around the globe—regardless of country, region, race/ethnicity, socioeconomic status, or culture. Expectations are that additional etiologic relationships will emerge over the coming decades, influenced by ever-evolving populations, ecology, and economies as well as by advances in science and technology (*1**,**2*). The true potential to avoid or minimize chronic disease by preventing or treating infections may yet be substantially underestimated.

Controlling infectious diseases remains paramount to the health and well-being of persons and populations worldwide. The breakdown of public health and prevention measures leads to the resurgence of old and new microbial threats. Nevertheless, implementing and maintaining infection control measures is shifting disease patterns, so that today chronic diseases represent the major health burden of established economies (>90 million people in the United States) and are a rapidly growing burden in developing economies (http://www.cdc.gov/nccdphp/overview.htm) (*3*). This fact implies that preventing or mitigating chronic diseases of infectious etiology could have considerable positive impact on global and domestic health. Add to this the potential benefits of minimizing infections that influence the morbidity of preexisting chronic conditions. The result is a tremendous opportunity to reduce long-term illness and disability worldwide by maximizing infection prevention and control.

In this perspective, we focus on (non-HIV) infectious determinants of chronic diseases, in which >1 infectious agent(s) causes, precipitates, or drives the chronic disease or its long-term sequelae. Expanding on previously published discussions (*4**–**7*), we outline the causal connections and reasons for their emergence, describing the breadth of the field and the diverse pathways from microbial exposure to chronic disease. Lastly, we present a complex systems framework for the multifactorial interactions that often lead to long-term sequelae, citing current and emerging opportunities for research to prevent chronic diseases of infectious etiology and discussing the potential impact of these benefits.

## Infectious Disease–Chronic Disease Connections

For centuries, physicians and scientists hypothesized that infection might explain some chronic syndromes. Proof, however, lagged behind speculation. A paucity of tools to detect many agents and the challenges of linking past infection—sometimes decades in the past—with present chronic illness perpetuated the idea that most infectious diseases are acute illnesses, and that chronic diseases have noninfectious causes. By the latter third of the 20th century, however, exceptions to this dogma began to emerge. For example, hepatitis B virus (HBV) infection came to explain a large proportion of chronic liver disease (CLD) and hepatocellular carcinoma (HCC) in areas of endemic infection (*8*) (http://www.cdc.gov/ncidod/diseases/hepatitis). However, it was the discovery that *Helicobacter pylori* can induce gastric inflammation that truly transformed conventional thinking about the noncommunicable nature of many chronic conditions (*9*); in recognition of this groundbreaking achievement, Marshall and Warren were awarded the Nobel Prize in Physiology or Medicine 2005. Researchers have subsequently demonstrated that eradication of *H. pylori* can cure most cases of peptic ulcer disease, a chronic condition long attributed to noninfectious factors such as stress, diet, smoking, and family history (*7**,**9**,**10*). Today, scientists and physicians widely recognize the plausibility of infectious agent origins for chronic diseases.

The causal relationships fall into 3 basic categories. First, an infectious agent produces chronic illness or long-term disability through progressive tissue pathology or organ decompensation (e.g., HBV-associated CLD and HCC), attributable to direct effects of persistent infection (e.g., transformation of host cells, tissue invasion); or) immune response to the persistent infectious agent; or ongoing immune response after the infectious agent(s) is cleared. Second, the initial stages of infection cause permanent, lifelong deficits or disability (e.g., poliovirus-induced permanent paralysis). Third, infection indirectly predisposes a person to chronic sequelae (e.g., maternal infection during pregnancy leads to preterm delivery that, with or without infection of the infant, increases the child's risk for chronic neurologic and pulmonary deficits). Together, these diverse relationships create a cascade of opportunities to reduce the impact of chronic disease by interrupting infection before the outcome is irreversible.

**Figure 1:**
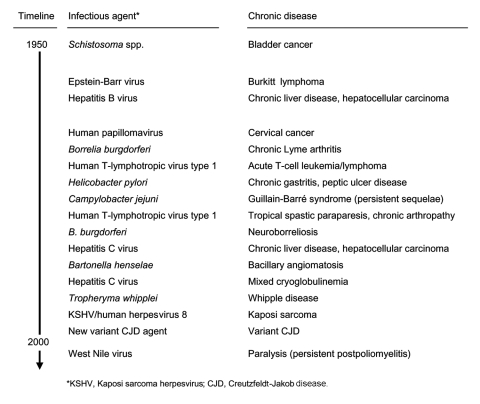
Emergence timeline for infectious determinants of chronic diseases.

Stimulated by changing scientific perceptions, the advent of polymerase chain reaction (PCR) and other molecular techniques, and advances in immunology and culture methods, a succession of discoveries from 1975 to 1995 greatly expanded the number of recognized infectious determinants of chronic diseases (Figure 1). We now know that HBV and hepatitis C virus (HCV) infections account for most CLD and HCC cases worldwide (*8*). In fact, HCC was the first recognized vaccine-preventable cancer (through HBV immunization). Blood donor screening, along with programs to prevent HBV and HCV transmission, now further reduces the risk for CLD and HCC (http://www.cdc.gov/ncidod/diseases/hepatitis) (*11**–**13*).

Today, immunization against human papillomavirus (HPV) promises to make cervical cancer—the second leading cause of cancer mortality in women worldwide—the next vaccine-preventable malignancy (*3*). Until now, cervical cancer prevention has hinged on early detection and ablation of precancerous and malignant lesions through lifelong Papanicolaou cervical smear screening of all women. While successful where economically feasible, this strategy does not address the infectious etiology of cervical cancer; studies associate HPV with 90% to 99.7% of malignant lesions (high-risk viral subtypes HPV-16 and HPV-18 with 65% to 70% of lesions), and HPV-induced oncoproteins are implicated in the pathway from infection to malignancy (*14**,**15*).

Microbes also cause nonmalignant chronic diseases. For example, *Borrelia burgdorferi* infections can result in chronic Lyme arthritis. In the absence of that discovery, an infectious portion of chronic inflammatory arthritis might still be categorized as a noninfectious autoimmune syndrome; *B. burgdorferi* and *B. garinii* infections also induce the chronic central nervous system manifestations of neuroborreliosis (*16**,**17*). These examples illustrate only a few of the numerous causal associations identified over the past 50 years; yet even they forecast the possibility that many other chronic conditions await the identification of infectious determinants.

Although the pace of discoveries has slowed over the past decade, at least 13 of the ≈39 most recently described infectious agents induce at least 1 distinct chronic syndrome (*1**,**13**,**16**,**18**–**20*). Most recently, a poliomyelitislike paralysis following West Nile virus infection expanded the list (*20*). With ample precedent, researchers, clinicians, and veterinarians can anticipate that infectious determinants of chronic diseases will continue to emerge.

## Reasons for Emergence

Evolving ecology and changing human behavior, such as migration, recreation, work, and culture, influence human exposures to the infectious determinants of chronic as well as acute illnesses (*1**,**2*). Microbial virulence factors, wildlife behavioral traits, zoonotic infections, and the environment all converge to determine both the infectious capacity of potential pathogens and the likelihood of human exposure. Superimposed on human genetics and biology, the milieu shapes individual and population risk profiles for the causal infections agents and their chronic sequelae (*7**,**14**,**21*).

Over recent years, the powerful tools of molecular biology, particularly PCR, plus advances in immunologic and other techniques, have exposed new causal links by detecting difficult-to-culture and novel agents in chronic disease settings. Microbes can now be irrefutably linked to pathology without meeting Koch's postulates, Hill's epidemiologic criteria, or even the revised criteria of Hill and Evans (*22*). For example, applying recombinant immunoscreening for the first time, investigators cloned the previously undescribed agent of most transfusion-associated (non-A, non-B) hepatitis and the cause of a major portion of chronic hepatitis, HCV (*23*). Innovative sequence-based analysis (broad-range PCR) and phylogenetic relationships finally identified *Tropheryma whipplei* as the elusive microbial source of Whipple disease (*19**,**22*). Improved culture techniques subsequently facilitated propagation of the bacterium. Now evidence confirms neurologic and ocular manifestations of this chronic gastrointestinal syndrome. Representational difference analysis identified the viral cause of Kaposi sarcoma (KS) in HIV-positive gay men (*24*). Later, researchers also linked the KS-associated herpesvirus to endemic or classic KS in the absence of HIV infection.

Today, technical advances boost the armory of detection tools available to uncover new infectious etiologies of chronic diseases, including the following: broad-range amplification of bacterial ribosomal targets, gene expression arrays (microarrays) that detect microbes or characterize host response to specific agents, degenerate probe screens for families or groups of viruses, mass spectrometry, electron microscopy, enhanced antigen and antibody detection techniques, and growth-promoting factors that improve microbe cultivation (*1*). The highly successful sensitivity of these tools, however, can be a double-edged sword. Detecting an infectious agent, its nucleic acid, or other biomarkers of infection in the setting of chronic disease does not prove it caused disease. Neither does the presence of antibodies to pathogens, for immunoglobulin G signifies previous infection but not necessarily causation (*22*). This fact is particularly true for ubiquitous infections. For example, chronic Lyme disease, reactive arthritis, CLD or HCC, peptic ulcer disease, cervical cancer, and Chagas cardiomyopathy develop only in some of the many people infected with *B. burgdorferi*, *Chlamydia trachomatis* or *Salmonella* species, HBV or HCV, *H. pylori*, HPV, and *Trypanosoma cruzi*. In contrast, the inability to detect an agent in the setting of chronic disease does not rule out infectious etiology. Existing tools and methods may not be sensitive enough to link known agents with chronic disease, or they may be unable to detect as yet uncharacterized novel or emerging microbes. Diagnostic assays might not access intracellular, sequestered, or nonreplicating agents. Testing may occur too long after the exposure, particularly when years of pathology precede diagnosis of the chronic condition, or persistent immune response to an already cleared infectious agent accounts for chronic disease. Studies that focus on the wrong group of people or the wrong tissue cannot support or refute causality. In all these circumstances, a true infectious determinant might remain unidentified.

## Breadth of the Field

**Figure 2:**
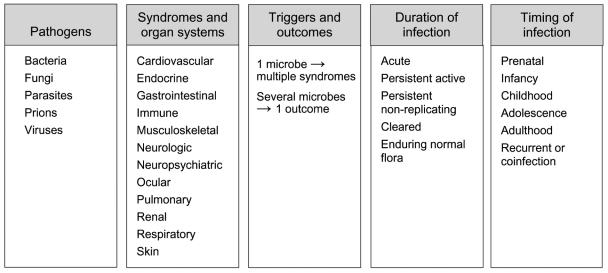
Infectious etiologies of chronic diseases.

A broad spectrum of infectious agents and their chronic outcomes compose this evolving field. Every organ system or tissue has been a target. Bacteria, fungi, parasites, viruses, and the recently discovered prions are all implicated, and as yet unidentified etiologic agents will likely be described over the coming years (Figure 2).

Already established causal associations prove that certain infectious agents evoke only 1 type of chronic pathology (e.g., poliovirus-induced persistent flaccid paralysis). Yet single agents can also produce multiple distinct syndromes in different organ systems. HBV-associated CLD, HCC, and polyarteritis nodosa, as well as HCV-associated CLD, HCC, mixed cryoglobulinemia, and arthropathy demonstrate this phenomenon (http://www.cdc.gov/ncidod/diseases/hepatitis) (*13**,**23**,**25**,**26*). So do 3 very different outcomes of human T-cell lymphotropic virus type 1 (HTLV-1) infection: acute T-leukemia/lymphoma, tropical spastic paraparesis/HTLV-1–associated myelopathy, and chronic arthropathy (*27**,**28*). On the other hand, disparate infections sometimes lead to 1 common chronic clinical syndrome, likely through converging pathogenic mechanisms (e.g., chronic HBV and HCV-related CLD or HCC; reactive arthritis following *Salmonella*, *Shigella*, *Klebsiella*, or *Chlamydia trachomatis* infections) (*21**,**23**,**25**,**26**,**29*).

A person's age at the time of infection—from intrauterine or perinatal, through childhood and adolescence, to adulthood and the elder years—may further influence the risk for chronic outcome. For example, perinatal HBV infection dramatically increases the risk of developing adult or pediatric CLD with or without HCC (*11**–**13**,**30*) (http://www.cdc.gov/ncidod/diseases/hepatitis). Recurrent infections or perhaps serial infections with certain agents might also determine a person's risk for chronic outcome.

Currently, the strength of causal evidence ranges from confirmed to speculative. Reproducible epidemiologic and laboratory data unambiguously establish that certain infectious agents directly lead to 1 or more distinct chronic outcome, globally or in unique populations. Animal models often illustrate the plausibility of human pathogenesis. Sometimes clinical trials and surveillance further demonstrate that preventing or treating the culprit infection(s) avoids or eliminates the long-term sequelae. Consider HBV-associated CLD. Sound scientific evidence now confirms that immunization and behavioral interventions prevent CLD and HCC by preventing infection and transmission (http://www.cdc.gov/ncidod/diseases/hepatitis) (*11**–**13*). Similarly, appropriate antimicrobial drug therapy can eliminate group A *Streptococcus* infections before rheumatic valvular disease develops and cure *H. pylori*–associated chronic gastritis and peptic ulcer disease (*7**,**9**,**10*). Unfortunately, the translation of infectious disease knowledge into programs that minimize pathology and the human suffering produced by chronic disease often lags, even when all evidence supports causality.

At the opposite end of the evidence spectrum, only preliminary or inconclusive findings, conflicting or inconsistent data, case series or small studies, anecdotal reports, or unreproduced single-source data support certain hypotheses. A lack of sensitive or specific detection assays, analyses that target the wrong tissue, or investigations that seek infectious agents too long after the initial infection might explain such observations. Suboptimal study designs also hamper the ability to reproduce or compare research results and to correctly infer causality. For example, if investigations examine only persons at low risk, only those at high risk, too few exposed or at-risk persons, or too many people not even at risk for the chronic outcome, then positive or negative findings can produce faulty conclusions. Studies lacking appropriate controls also convey uninterpretable results. On the other hand, evidence for or against an infectious etiology of chronic disease can change over time, influenced by new and sometimes contradictory findings, improved detection tools, and data interpretation. Onchocerciasis is an intriguing example of this fluidity. Infection with the filarial parasite *Onchocerca volvulus* is the long-established cause of river blindness. Recent evidence, however, suggests that the *Onchocerca* endosymbiont bacterium, *Wolbachia wuchereria*, may stimulate the pathogenic inflammation responsible for this tragic, preventable lifelong disability (*31*). If so, could *Wolbachia* also influence in whom *W. bancrofti*–associated lymphatic filariasis develops, potentially opening new therapeutic avenues to prevent this major cause of global disability (*32*)?

Despite the challenges, researchers continue to pursue elusive but plausible infectious agent origins of chronic syndromes such as systemic lupus erythematosus, rheumatoid arthritis and other inflammatory arthritides, Crohn disease, type 1 diabetes, multiple sclerosis, neuropsychiatric and developmental disorders, leukemias and lymphomas, and other malignancies (*33**–**44*). In concert, previously unrecognized long-term effects of known infectious agents continue to emerge.

## Range of Pathways

Directly or indirectly, infectious agents produce long-term outcomes through pathways that include acute infection, persistent active infection, persistent nonreplicating (latent) infection, immune response to an infectious agent that may not commonly be pathogenic, and malignant transformation. Direct tissue damage or genomic integration explain certain chronic sequelae, but an inflammatory immune response—one of the body's primary means to protect against infection—defines multiple established infectious causes of chronic diseases, including some cancers (*1**,**5**,**7**,**14**,**15**,**17**,**21**,**23**,**28**,**29*) (http://www.cdc.gov/ncidod/diseases/hepatitis). Inflammation also drives many chronic conditions that are still classified as (noninfectious) autoimmune or immune-mediated (e.g., systemic lupus erythematosus, rheumatoid arthritis, Crohn disease) (*33**–**35**,**38**,**40**,**41*). Both innate and adaptive immunity play critical roles in the pathogenesis of these inflammatory syndromes (*34**,**35*). Therefore, inflammation is a clear potential link between infectious agents and chronic diseases. Aberrant cellular and humoral responses to infections could launch the continuum from infection to long-term sequelae, consistent with the proposed damage-response framework (*6*)

Biofilms, or microbial communities that behave like biofilms, also represent potential, unrecognized stages in the pathways from infectious agent exposure to chronic disease. In both situations, cultures and even PCR results can be negative. For example, tympanic fluid cultures from animal models of chronic *Hamophilus influenzae* otitis media, associated with biofilms, are frequently negative (*45*); uropathogenic *Escherichia coli* can invade bladder epithelial cells to establish intracellular communities that behave like biofilms, evade immune surveillance, and produce sterile urine cultures (*46*). Similarly, imbalances within communities of normal gut flora or between commensals and pathogens residing in the gut are proposed to produce or exacerbate chronic syndromes such as Crohn disease (*35**–**37*). These observations suggest that novel and already characterized infectious agents are likely to determine a substantially greater—and potentially preventable—portion of chronic disease than yet realized. If so, upstream (earlier) primary and secondary prevention of infection will become opportunities to avoid irreversible or severe chronic disease across large populations.

**Figure 3:**
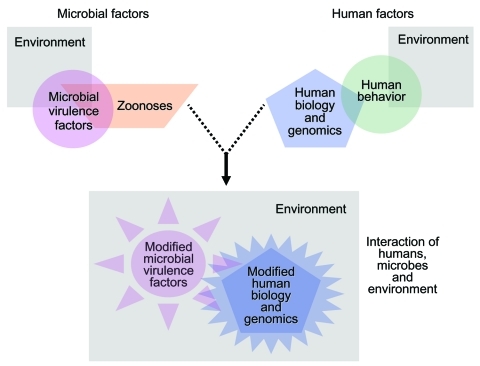
Complex systems framework, showing interaction of multiple factors leading to chronic sequelae of infections.

Frequently, the opportunity to identify new infectious determinants of chronic diseases may lie in the study of complex systems. Chronic diseases are often multifactorial, with established noninfectious risk factors. Yet infection actually defines more than a few of these conditions (e.g., cervical cancer, reactive arthritis). In such settings, complex systems, interactions between human, microbe, and the environment, tempered by time, determine microbial exposure, human infection, and the development of chronic sequelae (Figure 3). Simulating the balance, flux, and networks of multicomponent systems biology, many factors can converge to produce chronic disease, among them genetic susceptibility to infection or to adverse chronic outcome, duration of infection, co-infections, microbial factors, host microbial communities, age, micronutrient status, sex hormones, behavior-dependent exposures such as smoking and diet, chemical exposures, zoonoses, and the strength of an exposed person's immune response to an infectious agent(s) (*1**,**14**,**15**,**17**,**21**,**24**,**25**,**47**–**49*) (http://www.cdc.gov/ncidod/diseases/hepatitis). Human migration or travel, human-human interactions, evolving economies, political change, education, new medical interventions, changes in climate and ecology, and other factors further influence these complex systems.

Also diverging from the usual perceptions of causality, some hypotheses propose that infections may actually protect against certain chronic conditions; some microbial exposures may be critical to normal human immune development. Perhaps reduced or delayed exposure(s) to an infectious agent(s), or alterations in the balance of normal flora, increase a person's susceptibility to inflammatory conditions like asthma and Crohn disease (*37**,**50*).

## Current and Emerging Discovery and Prevention Opportunities

Chronic diseases do often stem from infections. Numerous causal associations are established, and progress in the field is certain to detect and confirm additional links. These developments should lead to new treatment regimens and public health programs that substantially reduce and even prevent chronic diseases worldwide, intervening before or during the early stages of disease to avoid or minimize the chronic sequelae of infections. If a mere 5% of chronic disease is attributable to infectious agents, in the United States alone 4.5 million of the 90 million people living with chronic disease might benefit from strategies designed to prevent or appropriately treat selected infections. Worldwide, the impact could be far greater. Avoiding exposure, reducing transmission, vaccinating to avert infection, and treating infection early could realize this prevention potential, dramatically reducing the global impact of chronic disease measured by disability-adjusted life years or other measures (*51*). The strategies must, however, build on sound scientific evidence.

Continued pathogen discovery and improved detection of infectious agents with sensitive, specific, reproducible assays are crucial to these efforts. In many settings, the systems biology approach will advance the timely recognition, characterization, and mitigation of infectious determinants of chronic diseases (*49*). Combining proteomics, genomics, microarrays, nanotechnology, and mass spectrometry with traditional detection tools such as histopathology may better confirm or refute hypotheses of causation, but only when applied to appropriate specimens from well-designed epidemiologic studies in the appropriate populations (*1*). Advances in information technology will be key to these efforts. The nature of chronic disease further demands longitudinal and prospective assessments since the symptoms of chronic disease may not appear until years after exposure to an infectious agent.

At present, cancers, autoimmune or immune-mediated diseases, and neurodevelopmental disorders are leading candidates for infectious agent origins. Yet other chronic conditions must also remain under consideration. Together, infectious determinants of chronic diseases offer a spectrum of research and prevention possibilities—opportunities that could substantially affect global health by reducing chronic disease worldwide. Not all chronic conditions will have infectious agent roots. Nevertheless, the broad prevention potential presented by these causal relationships has emerged as an important, cross-cutting clinical and public health issue, a result of the increased risk posed by newly recognized agents and changing population exposures as well as an increased appreciation for the causal links.
